# Гепатогенный сахарный диабет: обзор литературы и описание трех клинических случаев

**DOI:** 10.14341/probl13443

**Published:** 2025-05-20

**Authors:** М. В. Амосова, И. В. Полубояринова, П. В. Сальникова, К. Ю. Жеребчикова, В. В. Фадеев

**Affiliations:** Первый Московский государственный медицинский университет имени И.М. Сеченова (Сеченовский университет); Первый Московский государственный медицинский университет имени И.М. Сеченова (Сеченовский университет); Первый Московский государственный медицинский университет имени И.М. Сеченова (Сеченовский университет); Первый Московский государственный медицинский университет имени И.М. Сеченова (Сеченовский университет); Первый Московский государственный медицинский университет имени И.М. Сеченова (Сеченовский университет)

**Keywords:** гепатогенный сахарный диабет, сахарный диабет, цирроз печени, портальная гипертензия, портокавальный шунт, хронические заболевания печени

## Abstract

Гепатогенный диабет (ГепД) – это форма сахарного диабета, в основе патогенеза которой лежит первичное заболевание печени (как правило цирроз), осложнившееся развитием портальной гипертензии с образованием порто-кавальных шунтов. В развитии ГепД помимо традиционных факторов риска развития нарушений углеводного обмена большую роль играют патогенетические особенности заболеваний печени. Тем не менее, точный механизм развития гепатогенного диабета до конца не ясен, и ряд вопросов до сих пор остаётся открытым к обсуждению. Несмотря на то, что гепатогенный диабет имеет отчетливые патофизиологические и клинические особенности, в настоящее время он не рассматривается как самостоятельное заболевание. Вероятно, это связано с трудностями дифференциальной диагностики типа сахарного диабета из-за двунаправленного характера связи нарушений метаболизма глюкозы и хронических заболеваний печени. Известно, что сахарный диабет (СД) негативно влияет на развитие и прогрессирование хронических заболеваний печени (ХЗП) различной этиологии, их сочетание ассоциировано с худшими клиническими исходами в плане смертности, возникновения печеночной декомпенсации и развития гепатоцеллюлярной карциномы (ГЦК). К сожалению, ранняя диагностика и подбор оптимальной терапевтической стратегии лечения диабета могут быть затруднены из-за отсутствия установленных клинических рекомендаций, а также полиморбидности пациентов с ГепД.

## Abstract (en)

Hepatogenic diabetes (HepD) is a form of diabetes where the primary pathogenesis is a liver disease, usually cirrhosis, complicated by the development of portal hypertension with the formation of porto-caval shunts. In the development of HepD, in addition to traditional risk factors for carbohydrate metabolism disorders, the pathogenetic features of liver diseases play a significant role. However, the exact mechanism of HepD development remains unclear, and several questions are still open for discussion. Despite having distinct pathophysiological and clinical features, hepatogenic diabetes is currently not considered as an independent disease. This is likely due to the difficulties in differentiating between types of diabetes due to the bidirectional relationship between glucose metabolism disorders and chronic liver diseases. It is known that diabetes negatively affects the development and progression of chronic liver diseases of various etiologies, and their combination is associated with worse clinical outcomes in terms of mortality, the occurrence of liver decompensation, and the development of hepatocellular carcinoma (HCC). Unfortunately, early diagnosis and the selection of optimal therapeutic strategies for diabetes may be challenging due to the lack of established clinical guidelines and the presence of comorbidities in patients with HepD.

## АКТУАЛЬНОСТЬ

Гепатогенный диабет (ГепД), в отличие от сахарного диабета 2 типа (СД2), характеризуется отсутствием семейного анамнеза, взаимосвязи с избыточной массой тела, меньшей частотой развития микро- и макрососудистых осложнений, а также наличием выраженной инсулинорезистентности, что может вызывать определенные трудности в диагностике и лечении этого заболевания. Несмотря на наличие специфических причин развития нарушения работы печени, а также отчетливые патофизиологические и клинические особенности течения ГепД, этот тип диабета не выделяется в самостоятельное заболевание, в связи с чем является малоизученным состоянием в клинической практике. В настоящее время не существует единых алгоритмов по ведению пациентов, больных ГепД, что представляет собой существенную проблему и требует особого внимания. Понимание эпидемиологии, патофизиологии, клинических проявлений, лабораторной диагностики и лечения ГепД необходимо как для гепатологов, терапевтов, так и для эндокринологов.

## ВВЕДЕНИЕ

Влияние хронической гипергликемии на печень изучено достаточно. Известно, что декомпенсация углеводного обмена способствует нарушению функции гепатоцитов, снижению запасов в них гликогена, накоплению липидов, что ведет к развитию жирового гепатоза [1]. Однако результаты последних исследований говорят об обратном: о возможности нарушения гомеостаза глюкозы и о возникновении СД вследствие некоторых хронических заболеваний печени (ХЗП) [2–4].

Фактически сам СД2 в контексте метаболического синдрома (МС) может способствовать развитию неалкогольной жировой болезни печени (НАЖБП), которая может закончиться циррозом [5]. Кроме того, такой этиологический агент заболеваний печени, как вирус гепатита С (ВГС), может оказывать прямое диабетогенное действие с ранних, предцирротических стадий посредством нескольких, частично неустановленных механизмов, приводящих к развитию инсулинорезистентности и, возможно, также к нарушению функции β-клеток [6][7].

Однако при сосуществовании цирроза печени и СД, независимо от очередности их развития, они оказывают влияние друг на друга, и далеко не всегда можно с уверенностью говорить о циррозе в качестве причины развития диабета.

Влияние СД2 и гепатогенного диабета (ГепД) на клинический исход цирроза оценивалось в нескольких исследованиях [8]. Было показано, что наличие СД связано с повышенным риском развития осложнений и смертности среди пациентов с циррозом печени. ГепД может увеличить риск общих осложнений цирроза печени, таких как печеночная энцефалопатия, кровотечение из верхних отделов желудочно-кишечного тракта, спонтанный перитонит, асцит, а также риск прогрессирования цирроза, развитие рака печени и смертность от рака печени (табл. 1) [9]. Все это указывает на перспективность исследования клинического течения, диагностики и лечения ГепД.

**Table:** Таблица 1. Сравнительная характеристика СД и ГепД при ХЗП Примечание. ГПН — гликемия плазмы натощак; ФР — факторы риска; ЦП — цирроз печени; ПГТТ — пероральный глюкозотолерантный тест; МАЛА — метформин-ассоциированный лактат ацидоз; ССП — сахароснижающие препараты.

Критерии	Гепатогенный СД	СД2
Постановка диагноза	После диагноза ЦП	До цирроза печени
Начало заболевания	Субклиническое. Нормальная ГПН, HbA1c, может выявляться в ПГТТ	Явное. Повышение ГПН, HbA1c
Гипогликемия и МАЛА	Высокий риск	Низкий риск
Эффект от трансплантации	Ремиссия СД или снижение потребности в ССП	Отсутствует
Традиционные ФР	Реже представлены	Часто
Осложнения СД	Реже представлены	Часто
Осложнения заболевания печени	Выше, чем у лиц с ЦП без диабета	Выше, чем у лиц с ЦП без диабета
Смертность	Выше, чем у лиц с ЦП без диабета	Выше, чем у лиц с ЦП без диабета

В литературе распространенность СД среди пациентов с циррозом печени весьма вариабельна и колеблется от 20 до 70%. Такая высокая вариабельность данных в исследованиях связана с разными диагностическими критериями, стадиями и этиологией заболеваний печени. Вполне вероятно, что высокая распространенность диабета при циррозе печени связана с ранним появлением выраженной инсулинорезистентности в сочетании с недостаточностью функции бета-клеток, усугубляемой факторами, связанными с заболеванием печени.

Более поздние исследования с использованием современных критериев диагностики СД2 показали, что, основываясь на уровне глюкозы в плазме натощак (ГПН), отдельно или в сочетании с измерениями гликированного гемоглобина (HbA1c), распространенность СД у лиц с циррозом печени составляет около 30–40%. Однако было обнаружено увеличение распространенности нарушений углеводного обмена среди пациентов с циррозом печени, которым дополнительно проводился пероральный глюкозотолерантный тест (ПГТТ) вне зависимости от уровней ГПН и HbA1c. Таким образом, было показано, что уровни ГПН и HbA1c могут быть ложно нормальными у пациентов с циррозом печени в результате нарушения метаболизма глюкозы в печени, а также из-за сокращения продолжительности жизни эритроцитов вследствие гиперспленизма [10]. Эти данные указывают на необходимость проведения ПГТТ для диагностики СД у пациентов с циррозом печени даже при нормальных уровнях ГПН и HbA1c.

Тем не менее не у всех пациентов с СД и циррозом можно говорить о наличии истинного ГепД. Фактически у большинства пациентов СД существует еще до постановки диагноза цирроза печени.

ГепД можно определить как состояние нарушенной регуляции глюкозы, вызванное снижением функции печени в результате цирроза. Данное определение подразумевает, что СД развивается после возникновения цирроза печени.

## МЕХАНИЗМЫ РАЗВИТИЯ ГЕПАТОГЕННОГО ДИАБЕТА

Поскольку печень является одним из основных органов, участвующих в гомеостазе глюкозы, неудивительно, что у пациентов с ХЗП могут развиваться нарушения толерантности к глюкозе и ГепД.

Цирроз печени — потенциально возможная конечная стадия любого хронического заболевания печени. Цирроз характеризуется замещением нормальной печеночной ткани плотными соединительнотканными волокнами, что приводит к нарушению печеночного кровотока и снижению функции печени.

Нарушение печеночного кровотока приводит к повышению давления в воротной вене (портальной гипертензии). При повышении давления в воротной вене кровь направляется по анастомозам в обход печени. Портокавальные анастомозы в норме развиты слабо. Но при нарушении оттока крови по воротной вене могут значительно расширяться, благодаря чему снижается давление в системе воротной вены.

Коллатеральное кровоснабжение осуществляется не только по внутрипеченочным шунтам, но и по внепеченочным портокавальным анастомозам. Значительная часть крови при этом проходит через печень по перегородочным сосудам, минуя паренхиму. При формировании портокавального шунта все продукты обмена, минуя печень, сбрасываются в нижнюю полую вену, а инсулин, минуя печень, оказывается в системе нижней полой вены и циркулирует в системном кровотоке, что обуславливает развитие гиперинсулинемии, что в свою очередь способствует снижению сродства и числа рецепторов инсулина в тканях и приводит к формированию инсулинорезистентности [11].

При резистентности печени к действию инсулина происходит переключение процессов метаболизма: усиливается синтез и секреция в кровь глюкозы, начинается распад гликогена, а его образование и накопление в печени угнетается. При имеющейся саркопении, характерной для больных с различными заболеваниями печени, инсулинорезистентность будет выражена сильнее (рис. 1) [12].

**Figure:**
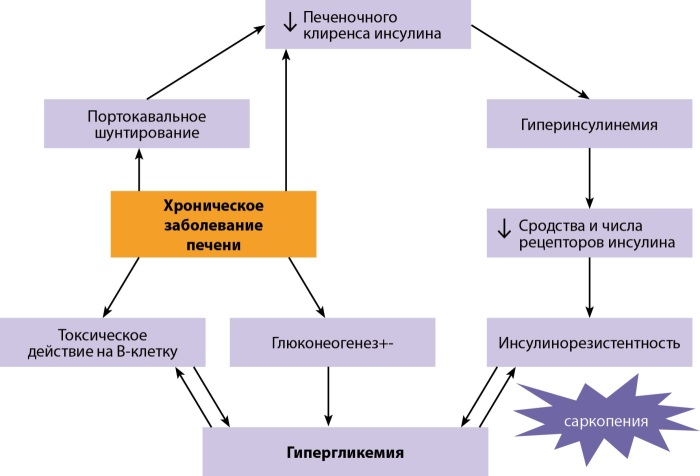
Рисунок 1. Упрощенная схема патогенеза гепатогенного сахарного диабета.

Характерной чертой гепатогенного диабета является то, что уровень инсулина в сыворотке значительно выше, чем при классическом СД2 [13].

С одной стороны, несколько исследований показали, что гиперинсулинемия возникает как следствие снижения печеночного клиренса инсулина из-за портально-системного шунтирования и дисфункции гепатоцитов [14], с другой стороны, существует мнение, что эти изменения — позднее явление, которое только усугубляет ранее существовавшую гиперинсулинемию [15].

Пациенты с более выраженным циррозом имеют более длительную продолжительность заболевания и, следовательно, более длительное воздействие повреждающих факторов на β-клетки, что может привести к более глубокой дисфункции островков. Однако пораженная печень может также оказывать «токсическое» воздействие на β-клетки за счет конечных продуктов гликирования, накапливающихся в системном кровотоке из-за невозможности их утилизации в печени (рис. 2) [16].

**Figure:**
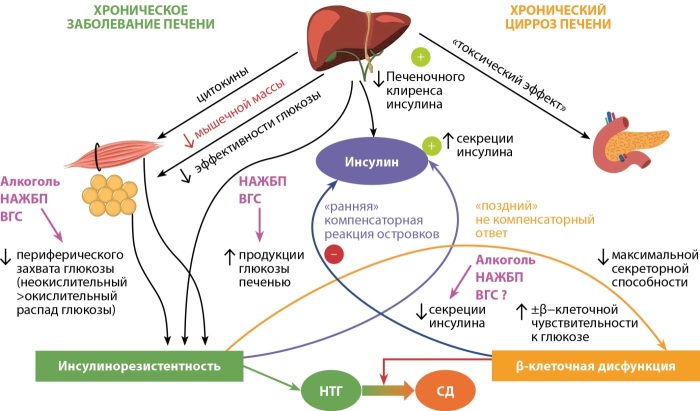
Рисунок 2. Расширенная схема патогенеза гепатогенного сахарного диабета.

Далее представлены три клинических случая пациентов с гепатогенным сахарным диабетом.

## ОПИСАНИЕ СЛУЧАЯ № 1

Пациентка Б., 35 лет (рост — 170 см, вес — 60 кг, ИМТ — 20,7 кг/м²), поступила в клинику эндокринологии с жалобами на слабость, жажду, повышение гликемии до 28,6 ммоль/л. Семейный анамнез отягощен: СД2 у бабушки. Из анамнеза заболевания известно, что в 29 лет пациентке диагностирован аутоиммунный цирроз печени, осложненный варикозным расширением вен пищевода (ВРВП) 2 ст., гипокоагуляцией, портальной гипертензией с последующим развитием портосистемных шунтов. Ранее получала терапию глюкокортикоидами, азатиоприном с положительным эффектом (нормализация лабораторных показателей). В 35 лет в ходе очередной госпитализации в гепатологическое отделение выявлено повышение гликемии до 31,4 ммоль/л, гиперосмолярность (303,2 мосмоль/л (285–295)), кетонурия отсутствовала, pH крови — 7,31, калий — 4,9 ммоль/л (3,5–5,0), HbA1c — 11,7%, С-пептид (базальный) — 500 пмоль/л (367–1467). Проводилась дробная инсулинотерапия, регидратационная терапия, коррекция электролитных нарушений. В дальнейшем пациентка переведена на базис-болюсную инсулинотерапию (ББИТ): Инсулин НПХ 36 ЕД в 08:00 и 20 ЕД в 22:00, Инсулин Р из расчета 1ХЕ:3,5ЕД перед основными приемами пищи (суточная доза 2,05/ЕД/кг). На этом фоне были достигнуты целевые показатели гликемии: натощак и перед основными приемами пищи 5,0–6,0 ммоль/л, постпрандиально до 8 ммоль/л, отсутствие гипогликемий. Признаков поздних осложнений СД выявлено не было.

Принимая во внимание молодой возраст, сохранный резерв бета-клеток, нормальную массу тела, наличие аутоиммунного гепатита, осложненного циррозом печени, наличие портосистемных шунтов, по всей вероятности, центральным звеном патогенеза является депортализация (венозная транспозиция), что в совокупности с приемом глюкокортикостероидов привело к прогрессированию инсулинорезистентности периферических тканей и развитию СД.

## ОПИСАНИЕ СЛУЧАЯ № 2

Пациентка Л., 49 лет (рост — 173 см, вес — 47 кг, ИМТ — 15,7 кг/м²) проконсультирована врачом-эндокринологом во время нахождения в отделении гепатологии и ОРИТ УКБ №2 ФГАОУ ВО ПМГМУ им. И.М. Сеченова в тяжелом состоянии. Известно, что длительное время наблюдается по поводу врожденного фиброза печени, портальной гипертензии, спленомегалии, ВРВП 1–2 ст. С 48 лет отметила появление болей в правом подреберье, сильно похудела (более 15 кг), стал увеличиваться в размерах живот, появились отеки ног. При компьютерной томографии органов брюшной полости — печень уменьшена в размерах, воротная вена — 22 мм, определяется анастомоз с варикозно расширенной сальниковой веной, правая и левая ветви окклюзированы, визуализируется реканализированная околопупочная вена диаметром 20 мм, впадающая в правую наружную подвздошную вену, визуализировались слабовыраженные спленогастральные, спленоренальные анастомозы. Также определялись расширенные легочные сосуды, спленомегалия и асцит (рис. 3). В это же время при обследовании выявлено повышение гликемии до 29 ммоль/л, диагностирован СД, начата инсулинотерапия. Назначенная инсулинотерапия включала в себя инсулин короткого действия внутривенно струйно под контролем гликемии. Отмечалась выраженная инсулинорезистентность, суточная доза инсулина составляла 150–200 ЕД (4,2 ЕД/кг), при этом меньшие дозы инсулина не позволяли достичь компенсации углеводного обмена.

**Figure:**
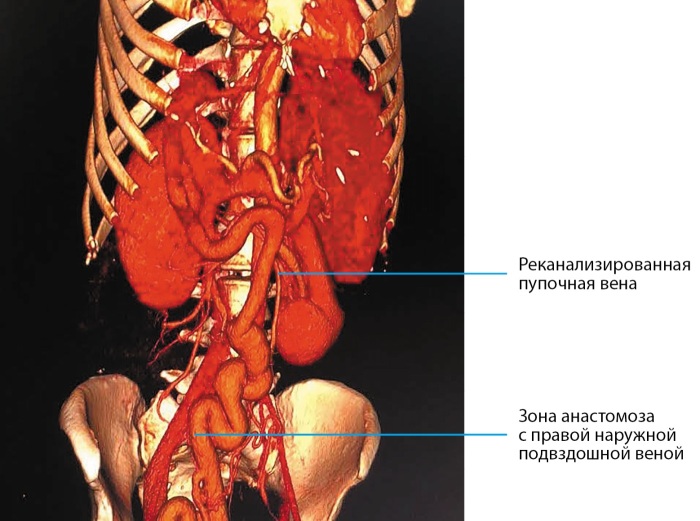
Рисунок 3. Компьютерная томография органов брюшной полости пациентки Л.: печень уменьшена в размерах, воротная вена — 22 мм, формируется анастомоз с варикозно-расширенной сальниковой веной, правая и левая ветви окклюзированы.

Тяжесть состояния пациентки была обусловлена гипергликемией, портальной гипертензией с портосистемным шунтом, а также энцефалопатией. Портальная гипертензия, по всей видимости, была ассоциирована с врожденным фиброзом печени. В пользу этого говорит отсутствие анамнеза хронического заболевания печени, сохранная функция печени, компенсированная сформировавшимся портосистемным шунтом. Предполагается, что в развитие гипергликемии, выраженной инсулинорезистентности, энцефалопатии значительный вклад вносило наличие портосистемного шунта.

Также на себя обращала внимание выраженная саркопения, которая является неблагоприятным прогностическим признаком и требует проведения нутриционной поддержки. Учитывая риски прогрессирования энцефалопатии при высокобелковом энтеральном питании, проводилась парентеральная нутриционная поддержка с применением специализированных аминокислотных смесей.

Учитывая выраженную гипергликемию (до 29 ммоль/л), с развитием гиперосмолярного состояния, гипераммониемию, вносящую вклад в развитие энцефалопатии и повторных эпизодов комы, пациентке была рекомендована ликвидация данного шунта.

Известно, что после проведения операции по закрытию шунта у пациентки отмечено значимое снижение потребности в инсулине, и в настоящее время она получает только базальный инсулин (инсулин детемир) в дозе 10 ЕД в 08:00 и 12 Ед в 22:00 (Ед/кг). На этом фоне показатели гликемии до 9 ммоль/л в течение дня, что может также указывать на развитие именно ГепД.

## ОПИСАНИЕ СЛУЧАЯ № 3

Пациентка Д., 34 года (рост — 168 см, вес —65 кг, ИМТ — 23 кг/м²). Семейный анамнез по СД отягощен. Из анамнеза известно, что в возрасте 29 лет (ИМТ — 27 кг/м²) отметила увеличение живота в объеме, отеки ног, отсутствие аппетита, жажду. При обследовании был выявлен цирроз печени неясного генеза класс В по Чайлд-Пью с синдромом портальной гипертензии. Также при проведении обследования было выявлено варикозное расширение вен пищевода 2 ст., гепатоспленомегалия, гиперспленизм (тромбоцитопения, лейкопения), реканализация пупочной вены, спленогастральные и спленоренальные анастомозы и асцит. У пациентки также имелась хроническая железодефицитная анемия тяжелой степени.

При обследовании было исключено инфицирование вирусами гепатита А, В, вирусом Эпштейн-Бара, цитомегаловирусом. Дважды HCV-РНК не выявлена, данных за гепатит С и аутоимунный генез цирроза печени не получено. По результатам эластометрии степень фиброза F4 по METAVIR. По данным биопсии печени: морфологическая картина монолобулярного цирроза печени низкой гистологической активности, стадия фиброза 4. При генетическом исследовании 47 генов, мутации в которых вызывают наследственные заболевания с преимущественным поражением печени, изменений, однозначно интерпретируемых как патогенные, не обнаружено. Также по результатам селективного скрининга данных за наследственные аминоацидопатии, органические ацидурии и дефекты митохондриального бета-окисления не выявлено. Выполнено секвенирование ДНК, выявлен не описанный ранее как патогенный вариант нуклеотидной последовательности в экзоне 1 гена ALG1 {chr16:5121868G>С}.

Через несколько месяцев после диагностики цирроза печени в рамках стационарного обследования выявлено повышение гликемии до 20 ммоль/л в течение дня, ацетон ±, HbА1с — 7,5%. Проконсультирована эндокринологом, инициирована базис-болюсная инсулинотерапия (инсулин гларгин 26 ЕД в 22:00, инсулин глилузин из расчета 1 ХЕ:3 ЕД перед завтраком, 1 ХЕ:2,5 ЕД перед обедом, 1 ХЕ:2,0 ЕД перед ужином (суточная доза инсулина 69 ЕД/кг, 1,06 ЕД/кг). По данным лабораторного исследования, уровень базального С-пептида — 936 пмоль/л (270–1730), С-пептид стимулированный (на фоне стандартной пищевой нагрузки) — 2730 пмоль/л. В последующем заболевание было интерпретировано как СД2, к терапии добавлен метформин 2000 мг в сутки, эмпаглифлозин 25 мг в сутки. На этом фоне гликемия — от 7 до 11 ммоль/л. Предпринимались попытки отмены инсулина ультракороткого действия с кратковременным эффектом. Неоднократно находилась на стационарном лечении по причине кровотечения из варикозно-расширенных вен пищевода, проводились эндоскопические лигирования.

В возрасте 33 лет в связи с прогрессированием заболевания проведена гепатэктомия, ортотопическая трансплантация трупной печени, атипичная резекция S3 трасплантата печени. В периоперационном периоде отменен метформин в связи с риском гипербилирубинемии, продолжена базис-болюсная инсулинотерапия. После проведенного оперативного лечения отмечено улучшение общего состояния, биохимическая ремиссия заболевания (аланинаминотрансфераза — 8 ед/л (10–35); альбумин — 42,1 г/л (35–52); аспартатаминотрансфера — 13 ед/л (0–35)). Гликемия — до 8 ммоль/л в течение дня.

После проведенной трансплантации печени пациентка поступила в эндокринологическое отделение с жалобами на тенденцию к низким значениям гликемии, при этом в питании себя не ограничивала. В клинике проведена коррекция сахароснижающей терапии: отменен аналог инсулина ультракороткого действия, постепенно снижена доза аналога инсулина длительного действия с последующей его отменой. Продолжена терапия препаратом из группы иНГЛТ-2 эмпаглифлозином в дозе 25 мг/сутки в сочетании с препаратом из группы иДПП-4 вилдаглиптином в дозе 100 мг/сутки. На этом фоне гликемия от 6,5 до 8 ммоль/л. Пациентка прошла обследование на предмет наличия поздних осложнений СД: выявлена диабетическая нейропатия: дистальный тип, сенсорная симметричная форма, диабетическая нефропатия, ХБП С2А2.

Принимая во внимание отягощенную наследственность (у матери, бабки, дяди по материнской линии СД), избыточную массу тела (ИМТ — 27 кг/м²), а также выраженную инсулинорезистентность, в дебюте состояние было расценено как СД2, пациентка получала базис-болюсную инсулинотерапию в сочетании с метформином и эмпаглифлозином. Однако после проведенной трансплантации печени существенно снизилась потребность в инсулине, в связи с чем инсулинотерапия была отменена. В настоящее время точный генез диабета остается не ясным, однако учитывая значимое снижение потребности в инсулине после трансплантации печени, разрешение инсулинорезистентности, возникшей вследствие депортализации кровотока, у пациентки, вероятнее всего, наличие гепатогенного сахарного диабета.

## ОБСУЖДЕНИЕ

В описанных выше клинических случаях центральным звеном патогенеза гипергликемии, вероятно, является развитие инсулинорезистентности, обусловленной первичным заболеванием печени — циррозом, с развитием портосистемных шунтов, анастомозов той или иной локализации и размеров.

При этом в большинстве случаев терапия СД зависит не от стажа заболевания и не от состояния бета-клеток поджелудочной железы, а именно от тяжести течения первичного заболевания. По мере прогрессирования заболевания печени усиливается и гипергликемия, поэтому ГепД можно рассматривать как маркер ухудшения функции печени.

В настоящее время отсутствуют стандартизированные руководства по лечению ГепД, и несмотря на то, что различные патофизиологические основы могут влиять на выбор тактики, лечение СД2 и ГепД проводят аналогичным образом. Ведение пациентов с ГепД затруднено из-за ограничений применения ряда сахароснижающих препаратов [17].

К сожалению, исследования, посвященные влиянию сахароснижающих препаратов, а также интенсивного контроля гликемии у пациентов с ХЗП отсутствуют или ограничены по размеру выборки и продолжительности наблюдения.

Существуют данные о благоприятном влиянии тиазолидиндионов (ТЗД) на течение заболеваний у пациентов с циррозом печени и СД, поскольку способствуют снижению инсулинорезистентности, а также способны нормализовать уровень аминотрансаминаз в крови [18]. В нескольких рандомизированных исследованиях было показано положительное влияние ТЗД на течение НАЖБП, в частности под влиянием пиоглитазона существенно улучшалась гистопатологическая картина печени [19][20].

Несмотря на обнадеживающие данные, ТЗД имеют ограниченное применение в силу ряда причин: риск развития сердечной недостаточности, периферические отеки и риск переломов. При этом противопоказанием для применения этой группы препаратов рассматривается повышение печеночных трансаминаз более чем в 2 раза.

Метформин в большинстве случаев не назначается пациентам с повышенной активностью печеночных трансаминаз, а также с хронической печеночной недостаточностью. Хотя несколько исследований продемонстрировали на фоне применения метформина снижение смертности и риска развития гепатоцеллюлярной карциномы при низком риске развития лактатацидоза. Данные последних рандомизированных контролируемых исследований и метаанализов не подтвердили наличие положительного влияния метформина на активность воспалительных изменений печени, не менялась и выраженность фиброза [21].

Обнадеживающие результаты были получены при терапии препаратами инкретинового ряда, включая агонисты рецептора ГПП-1 и ингибиторы ДПП4, которые имеют благоприятный фармакокинетический профиль у пациентов с ХЗП и улучшают течение НАЖБП [22]. Как правило, они безопасны для пациентов с ЦП, увеличивают мышечную массу и имеют низкий риск гипогликемии или увеличения веса [17].

В экспериментальных исследованиях на животных моделях было выявлено, что ингибиторы НГЛТ-2 обладают антифиброзным действием при стеатозе печени (подавление активности маркеров эндоплазматического ретикулярного стресса (GRP78,CHOP), концентрации ФНО-α, активности макрофагов и Т-клеток в белой жировой ткани и печени) [23]. С учетом их фармакокинетики, отсутствия метаболизирования в печени, а также таких благоприятных эффектов ингибиторы НГЛТ-2 могут быть кандидатами на роль препаратов, применяемых для лечения больных с ГепД [17]. Однако существуют данные о возможном усугублении течения саркопении на фоне лечения иНГЛТ-2, для получения полноценных и достоверных результатов требуется проведение дополнительных рандомизированных контролируемых клинических исследований [24].

Тем не менее у пациентов с терминальной стадией заболевания печени и тяжелой печеночной декомпенсацией инсулин является препаратом выбора.

Особый интерес представляет нормализация показателей углеводного обмена, уменьшение инсулинорезистентности на фоне закрытия шунтов или пересадки печени, что в очередной раз доказывает особый вклад печени в развитии диабета, а также подчеркивает необходимость выделения гепатогенного диабета в отдельный тип СД. По данным проведенных исследований, окклюзия шунта улучшает связанную с инсулинорезистентностью гиперинсулинемию за счет увеличения портального венозного кровотока, улучшения функции печени и, как следствие, увеличения печеночного клиренса инсулина у пациентов с циррозом печени [25]. Пересадка печени может приводить к снижению потребности в ССП или даже ремиссии диабета, однако в 30% случаев нарушения углеводного обмена могут сохранятся. Кроме того, известны случаи возникновения диабета de novo после трансплантации печени вследствие назначения иммуносупрессоров, глюкокортикоидов и т.д. [8].

В большинстве случаев формирующиеся у пациентов с циррозом печени шунты не оперируют, так как они оказывают компенсаторную функцию за счет обеспечения декомпрессии портальной системы, достаточной для регрессии варикозного расширения вен и профилактики пищеводно-желудочных кровотечений.

Вместе с тем саркопения, часто встречающаяся у пациентов с циррозом печени, считается прогностическим маркером выживаемости у пациентов с циррозом печени. Установлено, что у пациентов с саркопенией масса как скелетных мышц, так и жировой ткани увеличивалась после установки трансъюгулярного внутрипеченочного портосистемного шунта (95% ДИ: 1,2–7,8) [26]. Это, в свою очередь, сопровождалось улучшением клинического прогноза течения ХЗП.

Также было выявлено, что у пациентов с диабетом были выше риски развития таких состояний, как энцефалопатия, бактериальная инфекция, перитонит, по сравнению с пациентами без нарушений углеводного обмена [8].

Таким образом несмотря на то, что нарушения углеводного обмена не включены в наиболее широко используемые прогностические инструменты, а именно в шкалы Чайлд-Пью и MELD, наличие диабета является важным предиктором смертности при циррозе. Действительно, у пациентов с циррозом печени, находящихся в листе ожидания на трансплантацию, диабет, а не шкала Чайлд-Пью, был независимым предиктором смертности. Эти данные указывают на необходимость включения фактора наличия диабета в прогностическую оценку пациентов с циррозом печени, а также выделение отдельной нозологической единицы — гепатогенного диабета.

## ЗАКЛЮЧЕНИЕ

Таким образом, ГепД является недостаточно диагностируемым заболеванием, которое до сих пор плохо изучено и требует проведения дальнейших исследований. Необходимо выделить его из классического сахарного диабета 2 типа, возникающего у лиц с хроническим заболеванием печени, и идентифицировать лиц, страдающих этим заболеванием, в прогностических и терапевтических целях.

Требуются дополнительные исследования для выявления молекулярных механизмов, которые опосредуют токсическое влияние декомпенсации функции печени на β-клетки поджелудочной железы, а также для подтверждения положительного эффекта от окклюзии портосистемных шунтов, ортотопической трансплантации печени, а также влияния сахароснижающей терапии на течение хронических заболеваний печени.

Из-за неоднородности доступных исследований необходимы проспективные многоцентровые клинические испытания для дальнейшего изучения этой клинической проблемы. Необходима разработка клинических рекомендаций по ведению пациентов с ГепД для эффективной и своевременной диагностики патологического состояния и адекватной терапии. Достижение компенсации показателей углеводного обмена при ГепД важно не только для предотвращения типичных поздних осложнений диабета вследствие хронической гипергликемии, но и для предотвращения осложнений, связанных с циррозом печени.

## ДОПОЛНИТЕЛЬНАЯ ИНФОРМАЦИЯ

Источники финансирования. Исследование выполнено при финансовом обеспечении, лекарственном обеспечении, инструментальном обеспечении ФГАОУ ВО Первый МГМУ им. И.М. Сеченова Минздрава России (Сеченовский Университет).

Конфликт интересов. Авторы декларируют отсутствие явных и потенциальных конфликтов интересов, связанных с содержанием настоящей статьи.

Участие авторов. Все авторы одобрили финальную версию статьи перед публикацией, выразили согласие нести ответственность за все аспекты работы, подразумевающую надлежащее изучение и решение вопросов, связанных с точностью или добросовестностью любой части работы.

Согласие пациента. Пациенты добровольно подписали информированные согласия на публикацию персональной медицинской информации в обезличенной форме в журнале «Проблемы эндокринологии».

Благодарности. Авторы выражают благодарность сотрудникам эндокринологического терапевтического отделения №1 УКБ№2 ФГАОУ ВО Первый МГМУ им. И.М. Сеченова Минздрава России (Сеченовский Университет) за помощь в подготовке данного клинического случая.
